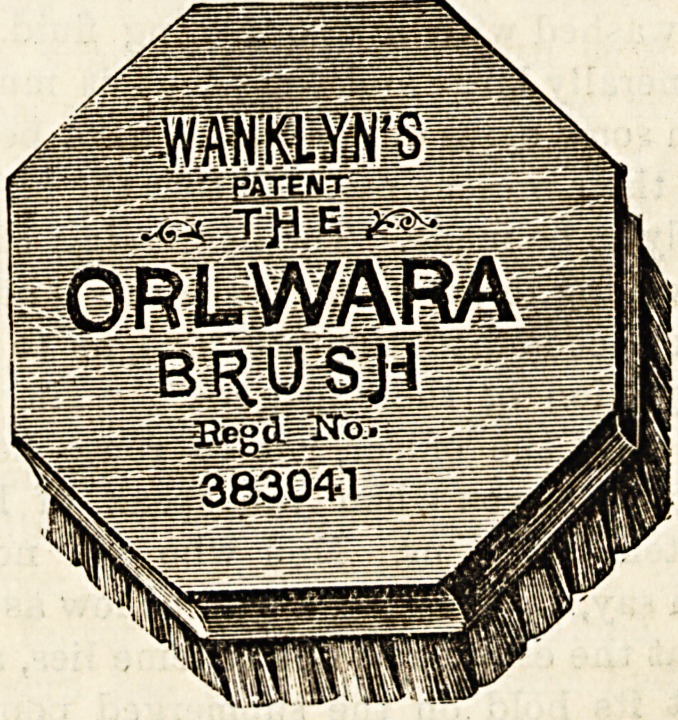# New Appliances and Things Medical

**Published:** 1902-10-18

**Authors:** 


### NEW APPLIANCES AND THINGS MEDICAL.

**[We shall be glad to receive at our Office, 28 & 29 Southampton Street, Strand, London, W.C., from the manufacturers, specimens of all new preparations and appliances which may be brought out from time to time.]**

### CANE SUGAR.

(Crosfields, Limited, Liverpool.)

From a chemical or physical point of view it is practically impossible to distinguish cane sugar from sugar which is derived from other vegetable sources, such as the beet. Physiologically there are possibly differences, and sentimentally these are strong reasons why we should in all cases use cane sugar from our own Colonies in preference to the manufactured beet sugar from our commercial rivals on the Continent. If it is impossible for experts to distinguish between these two varieties of sugar, it is of course useless for the public to attempt to do so, they can only rely on the good faith of the trader who undertakes to supply the genuine article. We believe that Crosfields, of Liverpool, can be invariably depended upon to supply absolutely pure cane sugar in the execution of orders. The three samples of their pure cane sugar, *i e.*, cubes, light brown, and fine grain, all appear of the highest quality.

### MILK CHOCOLATE.

(Cadbury Brothers, Limited. London Offices: 2 Rood Lane, London, E.C.)

The incorporation of milk with the other ingredients of chocolate is certainly a distinct advance in the manufacture of this confection, not only from the point of view of flavour and palatability but also because the addition of milk introduces elements of food which are of the greatest value from a dietetic point of view. The analysis of Cadbury's milk chocolate shows that it contains 30 per cent. of fat either in the form of cocoa-butter, or milk fat, 52 per cent. of sugar, and 10 per cent. of albuminoids. These figures prove that milk cocoa is a complete food, and owing to the high percentage of fat and sugar one particularly suitable for individuals who are exposed to great physical exertion in cold climates. For mountain climbers and Arctic explorers there could be no better food.

### THE "ORLWARA" NAIL BRUSHES.

(H. A. Wanklyn, 17 Manchester Avenue, Aldersgate Street, London, E.C.)

These new nail brushes are designed so as to obviate the wearing down of the bristles or fibres in the centre, which, owing to the shape invariably takes place in nail brushes, which are constructed on the old design of parallel rows of bristles set in a rectangular frame. In the Orlwara brushes the shape is round, octagonal, or square, and the bristle or fibre is distributed in such a manner that the wear falls as nearly as possible on all parts of the brush. From an economical point of view, it is clear that the more nearly round the shape of the brush, the larger will be the perimeter of the outside or useful rows of bristles in proportion to the central or useless ones. For this reason we specially commend the round pattern. The brushes which are made with bristles are greatly to be preferred to the cheaper ones made with fibre; the price in both cases is remarkably low.

## Figures and Tables

**Figure f1:**